# Association between high-density lipoprotein cholesterol and type 2 diabetes mellitus among Chinese: the Beijing longitudinal study of aging

**DOI:** 10.1186/s12944-021-01499-5

**Published:** 2021-07-17

**Authors:** Xue Cao, Zhe Tang, Jie Zhang, Haibin Li, Manjot Singh, Fei Sun, Xiaochun Li, Changwei Li, Youxin Wang, Xiuhua Guo, Deqiang Zheng

**Affiliations:** 1 Department of Epidemiology and Health Statistics, School of Public Health, Capital Medical University, Beijing, 100069 China; 2 Beijing Geriatric Healthcare Center, Xuanwu Hospital, Capital Medical University, Beijing, 100053 China; 3 Beijing Municipal Key Laboratory of Clinical Epidemiology, Capital Medical University, Beijing, 100069 China; 4 School of Medical and Health Sciences, Edith Cowan University, Joondalup, WA 6027 Australia; 5 Department of Epidemiology and Biostatistics, College of Public Health, University of Georgia, Athens, GA 30602 USA; 6 Department of Epidemiology, Tulane University School of Public Health and Tropical Medicine, New Orleans, LA 70118 USA

**Keywords:** Type 2 diabetes mellitus, Time-dependent variable, High-density lipoprotein cholesterol, Cox proportional-hazards model, Hazard ratio

## Abstract

**Background:**

Some previous studies on different populations have yielded inconsistent findings with respect to the relationship between levels of high-density lipoprotein cholesterol (HDL-C) and future type 2 diabetes mellitus (T2DM) incidence. This study was designed to gain further insight into this relationship through a cohort study with a 25-year follow-up duration.

**Methods:**

In total, 1462 individuals that were 55 years of age or older and were free of T2DM at baseline were enrolled in the present study. T2DM incidence among this study population was detected through self-reported diagnoses or the concentration of fasting plasma glucose. The data were derived from nine surveys conducted from 1992 to 2017. The correlation between HDL-C levels and the T2DM risk was assessed through Cox proportional-hazards model and proportional hazards model for the sub-distribution with time-dependent variables.

**Results:**

Over the follow-up period, 120 participants were newly diagnosed with new-onset T2DM. When research participants were separated into four groups on the basis for quartiles of their levels of HDL-C measured at baseline, and incidence of diabetes declined with higher baseline HDL-C levels at 12.60, 9.70, 5.38, and 5.22 per 1000 person-years, respectively. Adjusted hazard ratios (HRs) were 0.98 (95% confidence interval [CI]: 0.62–1.55), 0.48 (95% CI: 0.27–0.85) and 0.44 (95% CI: 0.25–0.80) for individuals with HDL-C levels within the 1.15–1.39, 1.40–1.69, and ≥ 1.70 mmol/L ranges relative to participants with HDL-C levels < 1.15 mmol/L. Multiple sensitivity analyses similarly revealed reduced risk of diabetes incidence with increased HDL-C levels. Incorporating the levels of HDL-C into a multivariate model significantly enhanced the overall power of the predictive model (*P* values were 0.0296, 0.0011, respectively, for 5- and 10-year risk of diabetes).

**Conclusions:**

Levels of HDL-C were independently and negatively associated with the risk of the new-onset T2DM among middle-aged and elderly Chinese.

**Supplementary Information:**

The online version contains supplementary material available at 10.1186/s12944-021-01499-5.

## Background

Diabetes mellitus is an increasingly widespread form of chronic disease that affected an estimated 5.9 and 9.3% of the global population in 2007 and 2019, respectively, [1] and with incidence rates that forecast to further increase to 10.9% by 2045 [1, 2]. An estimated 11.2% of Chinese adults currently suffer from diabetes, [3] despite the fact that type 2 diabetes mellitus (T2DM) is among the highly preventable diseases [4]. Notably, T2DM is a key reason of global mortality [5, 6]. Identifying reliable interventions targeting modifiable risk factors is thus important to effectively controlling and preventing this insidious disease [7].

Lower levels of high-density lipoprotein cholesterol (HDL-C) are more prevalently detected within the context of hyperglycemia, and as a result, metabolic syndrome-related parameters including HDL-C levels are often considered when evaluating a given individual’s risk of T2DM [8–10]. However, data pertaining to the link between HDL-C levels and T2DM incidence have differed among previous studies. In studies of American or European populations, lower HDL-C levels have been reportedly linked to higher risk of diabetes, [11–13] whereas no such relationship was observed in prior studies of Chinese populations [14, 15]. In our prior study of a Chinese population, it was confirmed that increased ratios of triglyceride (TG) to HDL-C (TG/HDL-C) were linked to an elevated risk of T2DM incidence, [16] but whether this association is primarily driven by reductions in HDL-C levels remains to be determined. These inconsistencies have the potential to be linked to the underreporting of T2DM incidence rates and the extended interval between follow-up visits. Most prior studies have primarily analyzed associations between HDL-C levels measured at baseline and the future incidence of diabetes, despite the fact HDL-C levels vary continuously with the passage of time. These factors may result in studies that contradict one another or yield less robust conclusions. As such, it is essential that a prospective study with multiple follow-up visits should be performed to fully elucidate the link between levels of HDL-C levels and the new-onset diabetes. As such, this current study explored this association using data from a cohort study with nine follow-up visits over a 25-year period, enabling us to assess the relationship between time-dependent levels of HDL-C and the future T2DM risk.

## Methods

### Population

Data for the present study were derived from the Beijing Longitudinal Study of Aging (BLSA), which evaluated community-dwelling Chinese individuals who were 55 years and older as discussed in prior reports [16–18]. Briefly, the BLSA study population was selected with the goal of ensuring accurate representation via a sampling approach, namely three-stage stratification random-clustering that was based upon the administrative district, age-gender distributions, educational level, and neighboring units, with administrative districts including the Huairou County (extended suburb, mountain) and Xuanwu District (urban), Daxing County (suburb, rural) being selected from among the 18 administrative districts of Beijing.

A total of 2101 representative study subjects completed initial baseline health exams in 1992 were enrolled in this study, with follow-up questionnaires being completed in 1994, 1997, 2000, 2004, 2007, 2009, 2012, and 2017, and with laboratory examinations being completed in 1992, 2000, 2009, 2012, and 2017. According to the exclusion criteria of this current study, research participants with a baseline diabetes diagnosis or baseline concentration of fasting plasma glucose ≥7.0 mmol/L were excluded (*n* = 246), as were individuals with missing baseline HDL-C measurements or other laboratory examination data (*n* = 393). In total, 1462 participants were included in the final analyzes. All BLSA participants had approved written aware consent, and the Ethics Committee of Capital Medical University confirmed this research (No. Z2019SY008), which was conducted as per the Declaration of Helsinki.

### Collection of data

A standardized stadiometer and weight-scale were used to measure the weight of study subjects when they were not wearing shoes and were only wearing thin clothing. Participant body mass index (BMI) was determined by weight (kg)/height squared (m^2^). The blood pressure (BP) was assessed by analyzing the right arm of each participant following a rest period of a minimum of 5 min. All laboratory testing was conducted on venous blood samples collected following overnight fasting in the central laboratory, Xuanwu Hospital, the Capital Medical University, which conducted all quality control and corresponding evaluations. Levels of TG, fasting plasma glucose (FPG), total cholesterol (TC), HDL-C and low-density lipoprotein cholesterol (LDL-C) were assessed with an automated biochemical analyzer (Hitachi 7150 Clinical/Chemistry Analyzer, HITACHI, Tokyo, Japan).

### Analyses of HDL-C levels and related covariates

Baseline levels of HDL-C were used to classify participants into four groups according to the first, the second and the third quartiles by combining the maximum and minimum values in the 1462 individuals included in this current study, with ranges of 0.52–1.14 mmol/L, 1.15–1.39 mmol/L, 1.40–1.69 mmol/L, and 1.70–5.03 mmol/L, respectively. Individuals from Daxing County and Huairou County were incorporated into a single group. Education levels were separated into two groups: secondary or higher, and primary or lower. Job types included heavy physical activity, light physical activity, and mental activity. Alcohol intake was considered to be mild, moderate, or heavy (< 50, 50–150, and > 150 mL/day). With respect to smoking, individuals were categorized as being ex-smokers, current smokers, or non-smokers. Dietary information pertaining to the consumption of eggs and staple foods was collected through questionnaires. Regular exercise levels were classified as low, moderate, or high (< 3, 3–10, and > 10 h/week, respectively. Hypertension was defined based upon a self-reported background of a hypertension diagnosis and/or on a systolic BP ≥ 140 mmHg and/or a diastolic BP ≥ 90 mmHg. Historical medical utilization of antihypertensive medications and drugs for treating cardiovascular disease was also obtained.

### Diagnosis of T2DM incidence

T2DM incidence among study subjects was detected based upon self-reported diagnosis or an FPG level ≥ 7.0 mmol/L (126 mg/dl) [19, 20]. Research participants were monitored for the incidence of diabetes or death from baseline in 1992 to the end of the follow-up period by 2017. Mortality-related data were derived from family member interviews and death certificates. The duration of follow-up for each study subject was the period time between baseline and the first to occur of diabetes incidence death, or loss to follow-up.

### Statistical analysis

Continuous variables were described as follows: means ± standard deviation (SD), and a thorough comparison were made via Kruskal-Wallis H tests. Categorical variables were displayed as frequencies with percentages and were compared via χ^2^ test. Associations between the levels of HDL-C and the incidence of T2DM were compared utilizing multivariate Cox proportional-hazard models. The proportional hazards for the sub-distribution proposed by Fine and Gray (Fine-Gray model) was also used to further assess the relationship between the levels of HDL-C and the incidence of diabetes given the possible influence of death as competing risk events [21]. Cox proportional-hazard model and Fine-Gray model were implemented to quantify hazard ratios (HRs) or sub-distribution HRs (SHRs) [22]. Age, alcohol intake, smoking status, BMI, regular exercise, staple food consumption, egg consumption, TG, LDL-C, hypertension, antihypertensive drug use, and cardiovascular medication use were treated as time-dependent confounding variables, while gender, level of education, residence, job types, and baseline FPG concentration were analyzed as time-invariant variables in time-dependent Cox proportional-hazards model and Fine-Gray model. Four models with steps were utilized to assess the relationship between the levels of HDL-C and risk of future T2DM incidence. Mean values were earmarked to each class of HDL-C levels and were treated as continuous variables when performing trend testing.

The relationship between the HDL-C levels and the incidence of diabetes was additionally assessed using Cox proportional-hazards model and Fine-Gray model with baseline dynamic factor levels. Sensitivity analyses were also performed wherein participants that were ≥ 75 years old in 1992 were excluded. The correlation between average HDL-C levels during follow-up and T2DM HRs was examined via a restricted cubic spline regression approach. Using the fully adjusted model, subgroup analyses based upon gender were also conducted, with HDL-C being used to separate study subjects into two groups based upon baseline HDL-C levels in 1992, using a value of 1.40 mmol/L as a cut-off for separation. The analyses of Cox regression were then executed to explore the capability of HDL-C levels to estimate T2DM incidence, with the curves of receiver operating characteristic (ROC) being generated and the discriminatory utility of these predictive models being evaluated based upon the area under the ROC curve (AUC).

SAS 9.4 (SAS Institute, NC, USA) and R 3.5.1 (R Foundation for Statistical Computing, Vienna, Austria) were implemented to accomplish statistical analyzes, with a *P* value lower than 0.05 as the significance threshold.

## Results

### Characteristics of participants

**Fig. 1:**
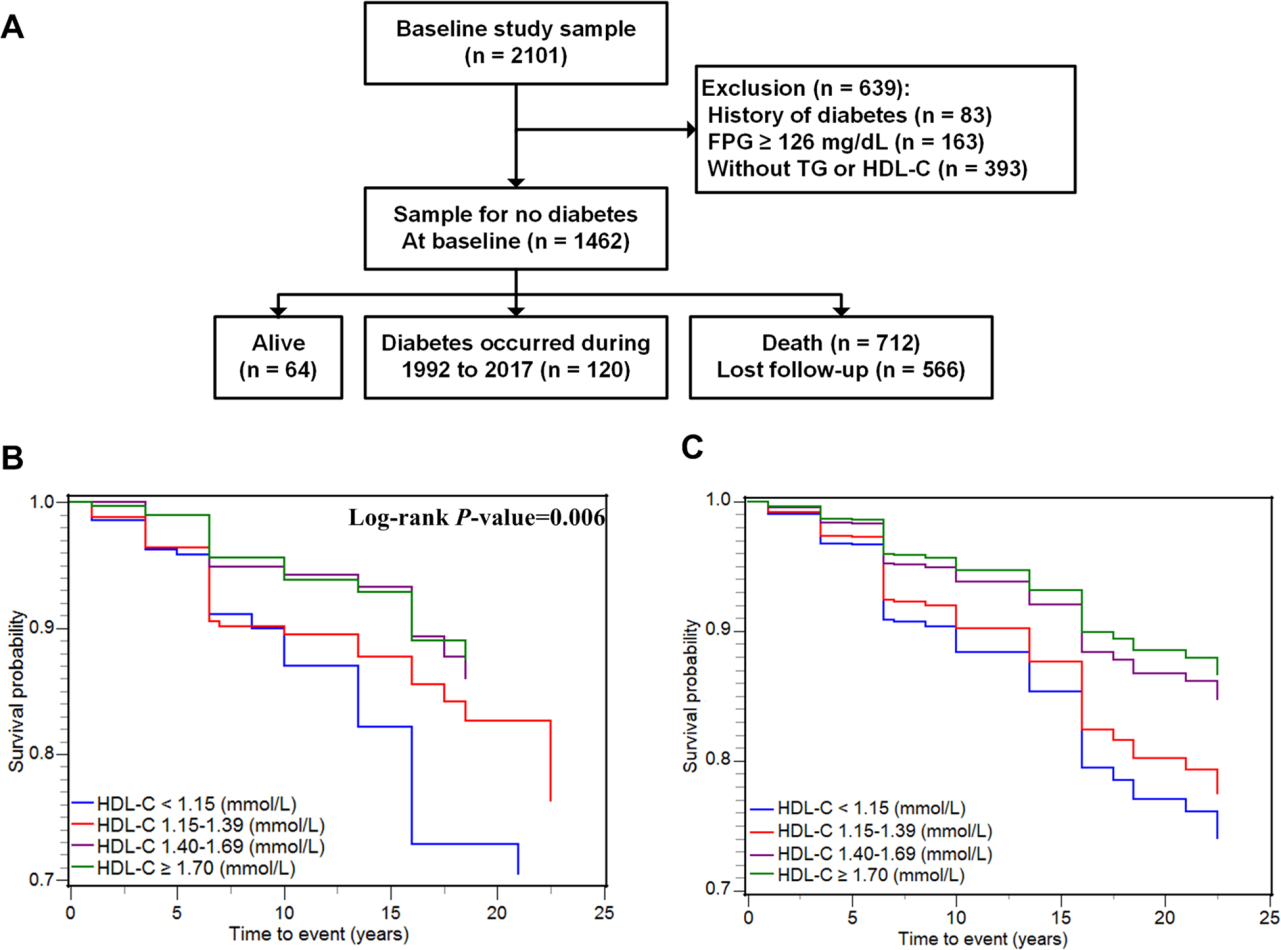
Flowchart demonstrating the participant selection process and survival curves corresponding to the four HDL-C quartiles. **(A).** Study participant selection flowchart. **(B).** Kaplan–Meier curve. **(C)**. Adjusted Cox regression survival curves used in Model IV

Over a median follow-up period of 8-years, 120 subjects developed new-onset T2DM, while 566 were lost to follow-up and 712 died. The mean baseline age of these 1462 participants was 68.81 ± 8.49 years. The schematic of the current research was illustrated in Fig. 1A, while participant characteristics summarized based upon HDL-C quartiles were demonstrated in Table 1. Roughly 80, 53, and 79% of the participants exhibited a baseline education level of primary or lower, were non-smokers, and consumed mild levels of alcohol, respectively. Over 60% of individuals in all four HDL-C quartiles exhibited hypertension at baseline, and those with greater levels of HDL-C exhibited lower BMI and TG levels values. Participant characteristics after 8 years of follow-up (2000) and at the last visit (2017) were respectively summarized in Table S1 and Table S2 in the supplementary materials. Over the follow-up of 14,157.04 person-years, the overall incidence of diabetes was 8.48/1000 person-years. These incidences declined with rising baseline HDL-C quartiles, with respective rates of which were 12.60, 9.70, 5.38, and 5.22 per 1000 person-years in each of these quartiles. Unadjusted Kaplan-Meier survival estimates for participants in these four baseline HDL-C quartiles were shown in Fig. 1B, with significant differences among groups as ascertained via log-rank test (*P* value < 0.001).

**Table 1:** Baseline features of research participants

Variables	High-density lipoprotein cholesterol (mmol/L)				*P* value
	< 1.15 (*n* = 365)	1.15–1.39 (*n* = 361)	1.40–1.69 (*n* = 364)	≥ 1.70 (*n* = 372)	
Age, year	68.15 ± 8.29	69.21 ± 8.18	69.26 ± 8.89	68.6 ± 8.54	0.279
Male, n (%)	207 (56.71)	178 (49.31)	176 (47.31)	172 (47.25)	0.032
Residence, n (%)					0.022
Urban	201 (55.07)	206 (57.06)	193 (51.88)	229 (62.91)	
Rural	164 (45.93)	155 (42.94)	179 (48.12)	135 (37.09)	
Educational level, n (%)					0.081
Secondary or higher	86 (23.56)	81 (22.44)	61 (16.40)	74 (20.33)	
Primary or lower	279 (76.44)	280 (77.56)	311 (83.60)	290 (79.67)	
Job types, n (%)					0.078
Mental activity	73 (20.0)	72 (19.9)	59 (15.9)	67 (18.4)	
Light physical activity	93 (25.5)	98 (27.1)	116 (31.2)	127 (34.9)	
Heavy physical activity	199 (54.5)	191 (52.9)	197 (53.0)	170 (46.7)	
Alcohol intake, n (%)					0.040
Mild	39 (10.68)	42 (11.63)	46 (12.37)	60 (16.48)	
Moderate	326 (89.32)	319 (88.37)	326 (87.63)	304 (83.52)	
Heavy	39 (10.7)	42 (11.6)	46 (12.4)	60 (16.5)	
Smoking status, n (%)					0.3810
Non-smoker	190 (52.1)	197 (54.6)	205 (55.1)	182 (50.0)	
Ex-smokers	50 (13.7)	63 (17.5)	59 (15.9)	64 (17.6)	
Current smokers	125 (34.2)	101 (28.0)	108 (29.0)	118 (32.4)	
Staple food, g/day, n (%)					0.119
≤ 300	131 (35.9)	150 (41.6)	144 (38.7)	163 (44.8)	
350–450	131 (35.9)	125 (34.6)	129 (34.7)	129 (35.4)	
≥ 500 g	103 (28.2)	86 (23.8)	99 (26.6)	72 (19.8)	
Egg consumption, a day, n (%)					0.026
0	59 (16.2)	67 (18.6)	52 (14.0)	48 (13.2)	
1	200 (54.8)	205 (56.8)	212 (57.0)	184 (50.5)	
> 1	106 (29.0)	89 (24.7)	108 (29.0)	132 (36.3)	
Regular exercise, hours/week, n (%)					0.572
< 3	145 (39.73)	146 (40.44)	157 (42.2)	145 (39.84)	
3–10	151 (41.37)	151 (41.83)	141 (37.90)	163 (44.78)	
> 10	69 (18.90)	64 (17.73)	74 (19.89)	56 (15.38)	
BMI (kg/m^2^)	23.9 ± 3.78	23.64 ± 3.84	22.75 ± 3.76	22.27 ± 3.85	< 0.001
TG (mmol/L)	1.74 ± 0.92	1.55 ± 0.72	1.29 ± 0.57	1.28 ± 0.65	< 0.001
LDL-C (mmol/L)	3.00 ± 0.95	3.17 ± 0.97	2.88 ± 0.99	2.89 ± 0.99	< 0.001
FPG (mmol/L)	5.06 ± 1.01	5.10 ± 1.01	5.19 ± 1.01	5.33 ± 0.84	0.003
Hypertension, n (%)	233 (63.8)	226 (62.6)	31 (62.1)	223 (61.3)	0.910
Antihypertensive drug, n (%)	58 (15.9)	52 (14.4)	52 (14.0)	45 (12.4)	0.595
CVD medication, n (%)	57 (15.6)	61 (16.9)	43 (11.6)	46 (12.6)	0.131

BMI, body mass index; CVD, cardiovascular disease; FPG, fasting plasma glucose; LDL-C, low-density lipoprotein cholesterol; TG, triglycerides

### Associations between the HDL-C levels and T2DM risk

Time-dependent HDL-C levels were negatively associated with the risk of future T2DM incidence (Table 2). After adjusting for gender, age, education, job type, regular exercise, smoking status, alcohol intake, residence (Model I), BMI, hypertension, staple food consumption and egg consumption (Model II), LDL-C, TG, antihypertensive drug use, and cardiovascular medication use (Model III), and baseline FPG concentrations (Model IV) in multivariate analyses, similar decreased HRs were calculated with respect to the incidence of diabetes as a function of elevated levels of HDL-C across different models. Adjusted HRs for individuals with time-dependent levels of HDL-C in Q2 (1.15–1.39 mmol/L), Q3 (1.40–1.69 mmol/L) and Q4 (≥ 1.70 mmol/L) were 0.98 (0.62–1.55), 0.48 (0.27–0.85) and 0.44 (0.25–0.80) relative to individuals in Q1 (< 1.15 mmol/L) in Model IV. Therefore, the risks of diabetes incidence for participants in Q3 and Q4 substantially declined through roughly 50% relative to those in the lowest HDL-C quartile. This trend towards reducing the T2DM risk was significant for the classes with elevated levels of HDL-C (*P* = 0.002), and SHRs yielded similar results (Table 2). In Q2, Q3, and Q4, HDL-C levels were still strongly correlated with the risk of diabetes, with SHRs of 1.07 (0.68–1.67), 0.52 (0.29–0.92) and 0.52 (0.30–0.91), respectively, relative to Q1 (Table 2).

**Table 2:** Adjusted hazard ratios for incidence of T2DM by the groups of HDL-C levels in the analyses of regression with time-dependent variables

	HDL-C (mmol/L)				
	< 1.15	1.15–1.39	1.40–1.69	≥ 1.70	
No. of participants	365	361	372	364	
No. of diabetes cases	46	35	20	19	
Incidence/1000 person-years	12.60	9.70	5.38	5.22	
	Hazard ratio (95% confidence interval)				*P*-value
Time-dependent Cox regression					
I	1.00	0.96 (0.62–1.50)	0.46 (0.26–0.78)	0.37 (0.21–0.64)	< 0.001
II	1.00	1.00 (0.64–1.56)	0.49 (0.28–0.85)	0.45 (0.26–0.78)	0.001
III	1.00	1.04 (0.66–1.64)	0.54 (0.31–0.95)	0.49 (0.28–0.88)	0.006
IV	1.00	0.98 (0.62–1.55)	0.48 (0.27–0.85)	0.44 (0.25–0.80)	0.002
Time-dependent Fine-Gray model					
I	1.00	0.97 (0.63–1.50)	0.45 (0.26–0.76)	0.39 (0.23–0.67)	< 0.001
II	1.00	1.03 (0.67–1.58)	0.48 (0.28–0.83)	0.48 (0.28–0.82)	0.002
III	1.00	1.10 (0.70–1.73)	0.56 (0.32–0.99)	0.56 (0.32–0.97)	0.014
IV	1.00	1.07 (0.68–1.67)	0.52 (0.29–0.92)	0.52 (0.30–0.91)	0.008

BMI, body mass index; FPG, fasting plasma glucose; LDL-C, low-density lipoprotein cholesterol; HDL-C, high-density lipoprotein cholesterol; TG, triglycerides

I: Adjustments were made for gender, age, education, smoking status, alcohol intake, regular exercise, residence and job type

II: Model I along with hypertension, BMI, consumptions of staple foods and eggs

III: Model II along with LDL-C, TG, antihypertensive drug and cardiovascular medication use

IV: Model III along with FPG

### Sensitivity analyses

**Fig. 2:**
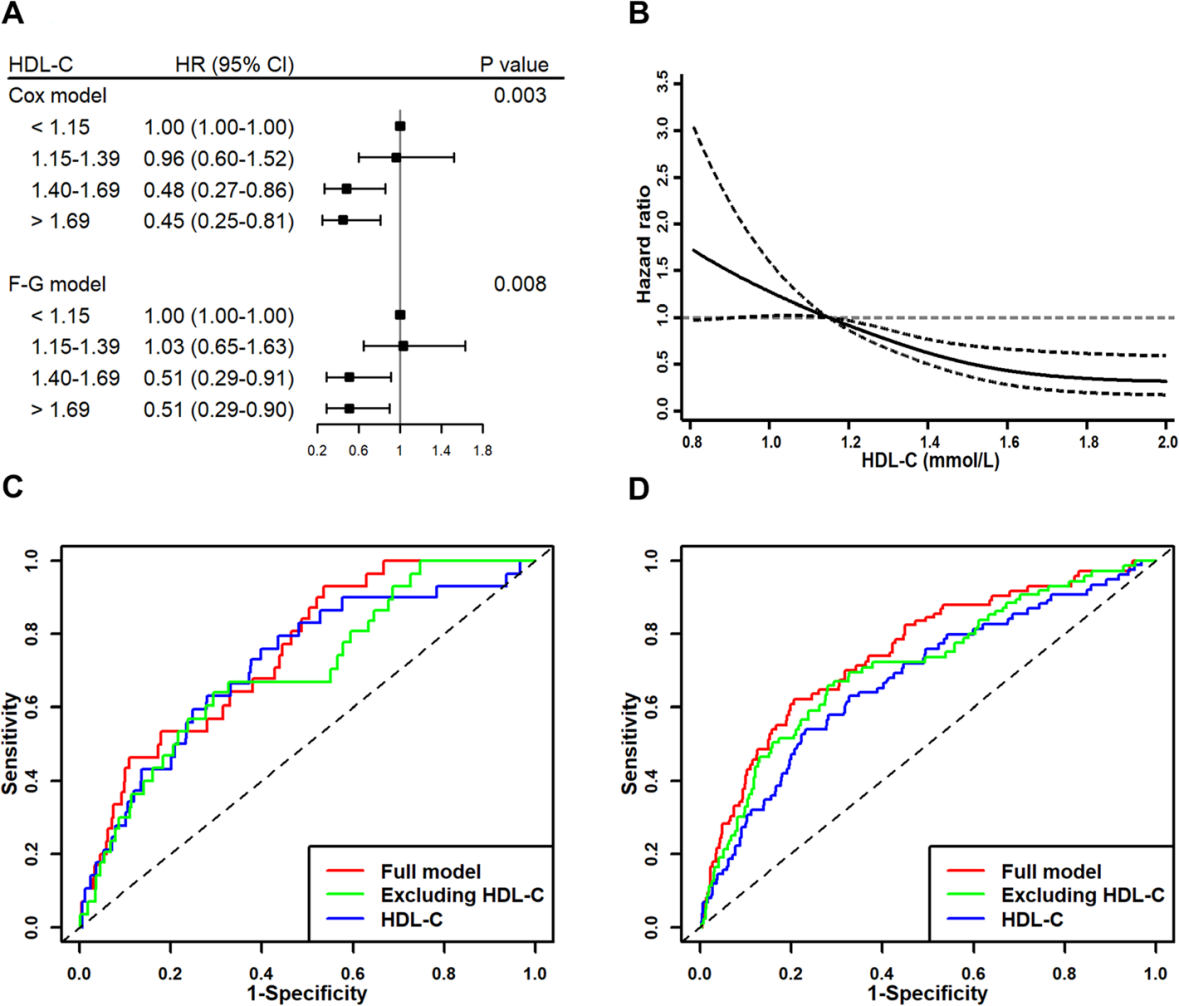
Sensitivity analyses of the association between HDL-C levels and T2DM risk. **(A).** Relationships between the levels of HDL-C and the risk of diabetes among individuals under the age of 75 (F-G: Fine-Gray model). **(B).** Associations between continuous levels of HDL-C and diabetes incidence in terms of dose-response, with 95% confidence intervals being indicated with dashed lines. **(C).** ROC curves based upon time-dependent Cox regression analyses used to predict 5-year risk. **(D).** ROC curves based upon time-dependent Cox regression analyses used to predict 10-year risk

A negative association of baseline HDL-C with T2DM incidence was also detected., and such that step elevation in HDL-C levels was correlated with reduced risks of T2DM (Table 3). Under the fully-adjusted Model IV, the HRs for the incidence of diabetes for individuals in Q2, Q3, and Q4 were, respectively, 0.75 (0.48–1.18), 0.49 (0.28–0.85) and 0.44 (0.25–0.77). Considerable differences in survival probabilities and increasing trends for adjusted survival curves were observed with increasing HDL-C levels across these four quartiles (Fig. 1C). Time-dependent Cox regression and Fine-Gray models yielded consistent results when participants who were > 75 years old at baseline were excluded from these analyses (Fig. 2A). Restricted cubic spline Cox regression analyses performed using average HDL-C levels during follow-up for participants similarly revealed lower HRs of diabetes with increased HDL-C levels (Fig. 2B). The subgroup analysis yielded similar results across different gender, with HRs for men and women of 0.48 (0.25–0.92), 0.49 (0.28–0.83), respectively (Fig. S1 in the supplementary materials).

**Table 3:** Adjusted hazard ratios (95% confidence interval) of T2DM incidence conforming to baseline levels of HDL-C

Model	HDL-C (mmol/L)				*P* value
	< 1.15	1.15–1.39	1.40–1.69	≥ 1.70	
Cox regression					
I	1.00	0.76 (0.49–1.19)	0.44 (0.26–0.76)	0.39 (0.23–0.67)	< 0.001
II	1.00	0.80 (0.51–1.25)	0.49 (0.29–0.84)	0.44 (0.25–0.77)	0.002
III	1.00	0.79 (0.51–1.25)	0.54 (0.31–0.94)	0.48 (0.27–0.84)	0.008
IV	1.00	0.75 (0.48–1.18)	0.49 (0.28–0.85)	0.44 (0.25–0.77)	0.004
Fine-Gray model					
I	1.00	0.77 (0.50–1.19)	0.43 (0.26–0.72)	0.39 (0.23–0.67)	< 0.001
II	1.00	0.82 (0.53–1.26)	0.47 (0.28–0.80)	0.45 (0.27–0.78)	0.003
III	1.00	0.82 (0.53–1.27)	0.53 (0.31–0.90)	0.49 (0.29–0.85)	0.009
IV	1.00	0.78 (0.50–1.22)	0.48 (0.28–0.82)	0.46 (0.27–0.79)	0.005

BMI, body mass index; FPG, fasting plasma glucose; HDL-C, high-density lipoprotein cholesterol; LDL-C, low-density lipoprotein cholesterol; TG, triglycerides

I: Adjustments were made for gender, age, education, smoking status, alcohol intake, regular exercise, residence and job type

II: Model I along with hypertension, BMI, consumptions of staple foods and eggs

III: Model II along with LDL-C, TG, antihypertensive drug and cardiovascular medication

IV: Model III along with FPG

### Assessment of the predictive utility of HDL-C levels

A univariate Cox proportional hazard model only including levels of HDL-C was constructed which revealed HDL-C levels to be a significant predictor of T2DM incidence, with values of AUC at 0.717 (95% CI 0.620–0.814) and 0.678 (95% CI 0.612–0.745) for 5- and 10-year risk of diabetes incidence, respectively (Fig. 2C and D). Furthermore, two Cox proportional hazard models incorporating significant factors including age, BMI, staple food consumption, HDL-C, and baseline FPG (Model IV) and TG used in previous diabetes prediction models, were built to examine the predictive utility of HDL-C levels. The full multivariate model incorporating HDL-C levels improved the AUC values from 0.698 (95% CI 0.606–0.791) and 0.714 (95% CI 0.650–0.779), respectively, to 0.749 (95% CI 0.672–0.826) and 0.753 (95% CI 0.692–0.813) for 5- and 10-year diabetes incidence, respectively (Fig. 2C and D). In addition, time-dependent ROC curves differed significantly between the full model and the model not incorporating HDL-C levels (*P* values were 0.0296 and 0.0011, respectively).

## Discussion

The results of this prospective analysis of community-dwelling Chinese adults in Beijing revealed an independent relationship between HDL-C and the risk of T2DM incidence. This negative correlation remained evident even when conducting multivariate competing risk analyses wherein death was treated as a competing outcome. The link between HDL-C and the risk of new-onset diabetes exhibited a dose-response relationship when treating HDL-C level as a continuous variable, and the incorporation of HDL-C into multivariate predictive models improved the overall predictive performance of these models. As such, all these data provide robust support for the conclusion that HDL-C were negatively correlated with the risk of future T2DM incidence.

These findings aligned well with prior evidence supporting a link between the levels of HDL-C and the future incidence of T2DM [11, 13, 23–25]. The Danish PREVENT study, which focused on a population of individuals with an average age of 49 years and without diabetes, determined that greater levels of HDL-C were protective against the onset of T2DM with an odds ratios (OR) and 95% CI of 0.55 (0.47–0.64) [11]. In the ASCOT-BPLA trial analyzing predictors of T2DM incidence among 14,120 participants, an HR of 0.72 (95% CI, 0.58–0.89) was reported for HDL-C levels increasing by one unit [23]. One longitudinal research of individuals in Framingham Offspring research similarly detected a negative relationship between HDL-C and T2DM incidence, having an OR of 0.96 (95% CI, 0.95–0.98) [25]. Two prior analyses conducted in China have yielded inconsistent findings. In one case, an analysis of a rural Chinese cohort observed no link between the levels of HDL-C as well as T2DM incidence, with an HR of 0.92 (95% CI, 0.70–1.19) in the group with the levels higher than the third quartile relative to the group with the levels less than the first quartile [14]. In the other case, a prospective analysis detected no link between HDL-C and T2DM risk (OR = 0.460; *P* = 0.189) [15]. However, in both of these studies, subjects were only evaluated at two follow-up time points during which medical histories were taken and examinations were completed, and these follow-up visits were at relatively late time points, respectively, at 7 and 15 years. The potential underestimation of T2DM development over such an extended interval may have adversely impacted efforts to gauge the true link between HDL-C and the new-onset T2DM risks. Two studies of Korean populations have yielded inconsistent conclusions. In line with our findings, one prospective cohort investigation on the basis of Korean National Health Insurance System enrolling 19,475,643 adults, with 5.13 years of the median follow-up period, found that lower HDL-C levels (men: < 40 mg/dL; women: < 50 mg/dL) to be correlated with a greater risk of T2DM [26]. In contrast, a retrospective cohort analysis of 5577 participants, during a 4-year follow up period, detected no significant link between these two variables, with an OR of 0.877 (95% CI, 0.753–1.021) for each one standard deviation increase of HDL-C levels [27].

The majority for these prior analyses, including the two studies of Chinese populations, primarily utilized a single measurement of HDL-C levels rather than adjusting for time-dynamic variables, thus potentially yielding inconsistent results owing to fluctuations in these levels as a function of time. In this present analysis, the HDL-C level was treated as a time-dependent variable, and considered the possible effects of other time-varying confounders that can possibly impact T2DM risk in order to accurately assess the correlation between HDL-C and the risk of future incidence of diabetes. Moreover, mortality as a competing event in order to decrease the bias associated with the right-censored treating of non-terminal events was processed. Importantly, analyses of this current study confirmed a link between HDL-C levels and the risk of diabetes incidence through both when treating HDL-C levels as a time-dynamic variable and when assessing these levels at baseline, confirming the robustness of conclusions. Few studies to date have explored the relationship between HDL-C levels and the risk of new-onset diabetes through both time-dependent and baseline analyses in a Chinese population.

A range of mechanisms may explain the link between increased levels of HDL-C and reduced risk of diabetes risk, including anti-inflammatory response mechanisms, improved insulin secretion and the uptake of glucose by the peripheral muscles [28–32]. One double-blinded placebo-controlled study observed significant improvements in plasma glucose levels in subjects that were intravenously administered reconstituted HDL-C (rHDL-C). Apolipoprotein (apo) A-I is a key HDL-C component that can promote AMPK pathway activation and thereby drive peripheral muscle glucose uptake [28, 29]. A series of in vitro analyses have provided experimental evidence for the ability of HDL-C to prevent or reverse reductions in insulin secretion associated with exposure to oxidized LDL-C, and one randomized controlled trial found that recombinant HDL infusion promoted AMPK pathway activation in the skeletal muscle of individuals with T2DM [29, 30]. In one analysis of rats and humans, HDL particles were shown to neutralize β-cell functional damage associated with oxidized LDL-C exposure via the JNK pathway [31]. Another mouse model study determined the link between lower HDL-C and the decreased uptake of glucose by peripheral skeletal muscle owing to a decrease in mitochondrial respiratory function within these cells [32]. Some prior publications have found that the protective impact of HDL-C in the context of T2DM risk was linked to changes in the function of pancreatic β-cell cells [33]. Atherogenic dyslipidemia and associated insulin resistance are thought to be driven in large part by increased hepatic free fatty acid flux, [27] leading to the liver-mediated secretion of particles containing apo-B [34]. A case-control analysis exploring associations among vitamin D, IFG, and T2DM further determined that HDL-C levels mediated 10.03% of the effects associated with vitamin D in males, while the ability of vitamin D levels to mediate the effects of HDL-C-C on T2DM in this same context was less evident [35].

### Strengths and limitations

This study has multiple important strengths. First, this was a standardized and well-designed cohort study with rigorous quality control, ensuring that the specimen of middle-aged and elderly individuals in China was as representative as possible. As such, conclusions in this study are robust and informative. Second, repeated HDL-C measurements in the study were treated as a time-variant variable to overcome the bias of regression dilution, and ensure that were more accurately examining the link between the levels of HDL-C and the future risk of new-onset T2DM. Third, competitive risk analyses were performed in which death was treated as a competing event to ensure that the conclusions were robust.

There are some limitations in this current study. For one, a lack of 2 h post-load glucose testing or fasting HbA_1c_ measurements suggests that some cases may have not been accurately diagnosed with T2DM. However, T2DM incidence was identified based on both self-reported diagnoses and fasting plasma glucose concentrations over a relatively short time period, such that the overall effect of T2DM underdiagnosis on this current study results can be decreased. Second, while this present study collected some data pertaining to dietary habits including the consumption of staple foods and eggs in this study, more detailed dietary details were not obtained. Although red wine intake was measured in this study, few participants (1.30%) consumed this form of alcohol, which was a luxury product in China in the 1990s. Third, by specifically focusing on middle-aged and elder population in Beijing, it is unable to determine whether the findings are applicable to other age groups or populations of other ethnics. As such, additional epidemiological and experimental research will be essential to deeper understand the link between HDL-C and the future risk of diabetes incidence.

## Conclusions

In conclusion, this study herein determined that the levels of HDL-C were independently and negatively linked to the risk of new-onset T2DM among middle-aged and elderly Chinese. Increased HDL-C levels were found to correlate with diminished T2DM risk over the 25-year follow-up period, suggesting that HDL-C levels may influence glucose metabolism. These findings may be of value by helping to guide the prevention of T2DM, and thus underscore the utility of lipid profiles as indices that can be used to predict diseases and to guide the design of clinical treatment plans.

## Supplementary Information

**Additional file 1 Supplementary Table S1.** Characteristics of study participants in 2000. **Supplementary Table S2.** Characteristics of study participants in 2017. **Supplementary Fig. S1.** Hazard Ratio (HR) and 95% confidence intervals (CI) with HDL-C on the risk of T2DM in the subgroup analysis

## Data Availability

The datasets used to support this study are available from the corresponding author on reasonable request.

## Abbreviations

HDL-C: high-density lipoprotein cholesterol
T2DM: type 2 diabetes mellitus
HRs: hazard ratios
TG: Triglycerides
BLSA: the Beijing Longitudinal Study of Aging
BMI: Body Mass Index
FPG: fasting plasma glucose
TC: total cholesterol
LDL-C: low-density lipoprotein cholesterol
CVD: cardiovascular disease
SD: standard deviation
SHR: Sub-distribution hazard ratio
ROC: receiver operating characteristic
AUC: the area under the curve
OR: odds ratios
AMPK: the AMP-mediated protein kinase pathway
apo: apolipoprotein
