# Supplementary material for: Association between high-density lipoprotein cholesterol and type 2 diabetes mellitus among Chinese: the Beijing longitudinal study of aging
Source: Lipids Health Dis. 2021 Jul 17;20:71. doi: 10.1186/s12944-021-01499-5 (PMC8286580; doi:10.1186/s12944-021-01499-5)
Supplement: Supplementary file 1 — Additional file 1 Supplementary Table S1. Characteristics of study participants in 2000. Supplementary Table S2. Characteristics of study participants in 2017. Supplementary Fig. S1. Hazard Ratio (HR) and 95% confidence intervals (CI) with HDL-C on the risk of T2DM in the subgroup analysis [file 12944_2021_1499_MOESM1_ESM.docx]

**Supplementary Material**

**Association between high-density lipoprotein cholesterol and type 2 diabetes mellitus among Chinese: the Beijing longitudinal study of aging**

Xue Cao^1^, Zhe Tang^2^, Jie Zhang^1,3^, Haibin Li^1,3^, Manjot Singh^4^, Fei Sun^2^,

Xiaochun Li^1, 3^, Changwei Li^5, 6^, Youxin Wang^1,3^, Xiuhua Guo^1,3^, Deqiang Zheng^1,3^

^1^Department of Epidemiology and Health Statistics, School of Public Health, Capital Medical University, Beijing 100069, China

^2^Beijing Geriatric Healthcare Center, Xuanwu Hospital, Capital Medical University, Beijing 100053, China

^3^Beijing Municipal Key Laboratory of Clinical Epidemiology, Capital Medical University, Beijing 100069, China

^4^School of Medical and Health Sciences, Edith Cowan University, Joondalup WA 6027, Australia

^5^Department of Epidemiology and Biostatistics, College of Public Health, University of Georgia, Athens, GA 30602, USA

^6^Department of Epidemiology, Tulane University School of Public Health and Tropical Medicine, New Orleans, LA 70118, USA

***Correspondence to:***

Deqiang Zheng; dqzheng@ccmu.edu.cn

| **Supplementary Table S1-** **Characteristics of study participants in 2000.** | | | | | | | |
| --- | --- | --- | --- | --- | --- | --- | --- |
| Variables | High-density lipoprotein cholesterol (mmol/L) | | | |  | |  |
|  | < 1.15  (n=95) | 1.15-1.39  (n=135) | 1.40-1.69  (n=167) | ≥ 1.70  (n=269) | *P* value |  |  |
| Age in years | 72.9±7.5 | 74.1±7.3 | 73.9±7.2 | 73.5±7.0 | 0.504 | |  |
| Male, n (%) | 43 (45.3) | 65 (48.1) | 83 (49.7) | 126 (46.8) | 0.901 | |  |
| Residence, n (%) |  |  |  |  | 0.019 | |  |
| Urban | 56 (58.9) | 73 (54.1) | 83 (49.7) | 114 (42.4) |  | |  |
| Rural | 39 (41.1) | 62 (45.9) | 84 (50.3) | 155 (57.6) |  | |  |
| Educational level, n (%) | |  |  |  | 0.700 | |  |
| Secondary or higher | 18 (18.9) | 28 (20.7) | 39 (23.4) | 65 (24.2) |  | |  |
| Primary or lower | 77 (81.1) | 107 (79.3) | 128 (76.6) | 204 (75.8) |  | |  |
| Job types, n (%) |  |  |  |  | 0.101 | |  |
| Mental activity | 18 (18.9) | 25 (18.5) | 40 (24.0) | 58 (21.6) |  | |  |
| Light physical activity | 20 (21.1) | 29 (21.5) | 38 (22.8) | 84 (31.2) |  | |  |
| Heavy physical activity | 57 (60.0) | 81 (60.0) | 89 (53.3) | 127 (47.2) |  | |  |
| Alcohol intake, n (%) |  |  |  |  | 0.057 | |  |
| Mild | 82 (86.3) | 112 (83.0) | 134 (80.2) | 203 (75.5) |  | |  |
| Moderate | 8 (8.4) | 15 (11.1) | 11 (6.6) | 32 (11.9) |  | |  |
| Heavy | 5 (5.3) | 8 (5.9) | 22 (13.2) | 34 (12.6) |  | |  |
| Smoking status, n (%) |  |  |  |  | 0.321 | |  |
| Non smoker | 56 (58.9) | 67 (49.6) | 94 (56.3) | 140 (52.0) |  | |  |
| Ex-smokers | 20 (21.1) | 30 (22.2) | 35 (21.0) | 75 (27.9) |  | |  |
| Current smokers | 19 (20.0) | 38 (28.1) | 38 (22.8) | 54 (20.1) |  | |  |
| Staple food, g/day, n (%) | |  |  |  | 0.027 | |  |
| ≤ 300 | 43 (45.3) | 62 (45.9) | 63 (37.7) | 121 (45.0) |  | |  |
| 350-450 | 30 (31.6) | 33 (24.4) | 48 (28.7) | 95 (35.3) |  | |  |
| ≥ 500 g | 22 (23.2) | 40 (29.6) | 56 (33.5) | 53 (19.7) |  | |  |
| Egg consumption, a day, n (%) | |  |  |  | 0.005 | |  |
| 0 | 17 (17.9) | 16 (11.9) | 12 (7.2) | 39 (14.5) |  | |  |
| 1 | 34 (35.8) | 49 (36.3) | 49 (29.3) | 63 (23.4) |  | |  |
| > 1 | 44 (46.3) | 70 (51.9) | 106 (63.5) | 167 (62.1) |  | |  |
| Regular exercise, hours/week, n (%) | | | | | 0.295 | |  |
| < 3 | 38 (40.0) | 51 (37.8) | 65 (38.9) | 83 (30.9) |  | |  |
| 3-10 | 36 (37.9) | 55 (40.7) | 63 (37.7) | 104 (38.7) |  | |  |
| > 10 | 21 (22.1) | 29 (21.5) | 39 (23.4) | 82 (30.5) |  | |  |
| BMI, (kg/m^2^) | 24.7±3.9 | 24.4±3.8 | 24.2±4.0 | 22.9±3.6 | <.001 | |  |
| TG, (mmol/L) | 2.34±1.57 | 1.71±0.86 | 1.41±0.74 | 1.19±0.64 | <.001 | |  |
| LDL-C, (mmol/L) | 2.58±0.79 | 3.00±0.82 | 3.00±0.89 | 2.98±0.84 | 0.001 | |  |
| FPG, (mmol/L) | 4.98±0.93 | 5.08±0.92 | 5.26±0.89 | 5.19±0.96 | 0.017 | |  |
| Hypertension, n (%) | 71 (74.7) | 104 (77.0) | 127 (76.0) | 184 (68.4) | 0.177 | |  |
| Antihypertensive drug, n (%) | 34 (35.8) | 41 (30.4) | 45 (26.9) | 63 (23.4) | 0.107 | |  |
| CVD medication, n (%) | 12 (12.6) | 29 (21.5) | 35 (21.0) | 57 (21.2) | 0.290 | |  |
| BMI, body mass index; CVD, cardiovascular disease; FPG, fasting plasma glucose;  LDL-C, low-density lipoprotein cholesterol; TG, triglycerides. | | | | | | | |

| **Supplementary Table S2- Characteristics of study participants in 2017.** | | | | | | | |
| --- | --- | --- | --- | --- | --- | --- | --- |
| Variables | High-density lipoprotein cholesterol (mmol/L) | | | |  | |  |
|  | < 1.15  (n=95) | 1.15-1.39  (n=135) | 1.40-1.69  (n=167) | ≥ 1.70  (n=269) | *P* value |  |  |
| Age in years | 84.3±2.6 | 86.5±3.6 | 86.1±4.5 | 84.1±3.1 | 0.107 | |  |
| Male, n (%) | 8 (72.7) | 5 (21.7) | 10 (66.7) | 8 (72.7) | 0.011 | |  |
| Residence, n (%) |  |  |  |  | 0.734 | |  |
| Urban | 4 (36.4) | 13 (56.5) | 7 (46.7) | 7 (46.7) |  | |  |
| Rural | 7 (63.6) | 10 (43.5) | 8 (53.3) | 8 (53.3) |  | |  |
| Educational level, n (%) | |  |  |  | 0.774 | |  |
| Secondary or higher | 5 (45.5) | 8 (34.8) | 6 (40.0) | 4 (26.7) |  | |  |
| Primary or lower | 6 (54.5) | 15 (65.2) | 9 (60.0) | 11 (73.3) |  | |  |
| Job types, n (%) |  |  |  |  | 0.490 | |  |
| Mental activity | 5 (45.5) | 5 (21.7) | 7 (46.7) | 4 (26.7) |  | |  |
| Light physical activity | 1 (9.1) | 3 (13.0) | 2 (13.3) | 4 (26.7) |  | |  |
| Heavy physical activity | 5 (45.5) | 15 (65.2) | 6 (40.0) | 7 (46.7) |  | |  |
| Alcohol intake, n (%) |  |  |  |  | 0.324 | |  |
| Mild | 8 (72.7) | 20 (87.0) | 9 (60.0) | 12 (80.0) |  | |  |
| Moderate | 1 (9.1) | 3 (13.0) | 4 (26.7) | 1 (6.7) |  | |  |
| Heavy | 2 (18.2) | 0 (0.0) | 2 (13.3) | 2 (13.3) |  | |  |
| Smoking status, n (%) |  |  |  |  | 0.028 | |  |
| Non smoker | 3 (27.3) | 18 (78.3) | 5 (33.3) | 9 (60.0) |  | |  |
| Ex-smokers | 7 (63.6) | 4 (17.4) | 6 (40.0) | 4 (26.7) |  | |  |
| Current smokers | 1 (9.1) | 1 (4.3) | 4 (26.7) | 2 (13.3) |  | |  |
| Staple food, g/day, n (%) | |  |  |  | 0.859 | |  |
| ≤ 300 | 9 (81.8) | 17 (73.9) | 10 (66.7) | 11 (73.3) |  | |  |
| 350-450 | 2 (18.2) | 5 (21.7) | 5 (33.3) | 3 (20.0) |  | |  |
| ≥ 500 g | 0 (0.0) | 1 (4.4) | 0 (0.0) | 1 (6.7) |  | |  |
| Egg consumption, a day, n (%) | |  |  |  | 0.143 | |  |
| 0 | 6 (54.5) | 15 (65.2) | 13 (86.6) | 10 (66.7) |  | |  |
| 1 | 5 (45.5) | 4 (17.4) | 1 (6.7) | 1. (13.3) |  | |  |
| > 1 | 0 (0.0) | 4 (17.4) | 1 (6.7) | 3 (20.0) |  | |  |
| Regular exercise, hours/week, n (%) | | | | | 0.300 | |  |
| < 3 | 6 (54.5) | 13 (56.5) | 7 (46.7) | 3 (20.0) |  | |  |
| 3-10 | 1 (9.1) | 4 (17.4) | 5 (33.3) | 10 (66.7) |  | |  |
| > 10 | 4 (36.4) | 6 (26.1) | 3 (20.0) | 2 (13.3) |  | |  |
| BMI, (kg/m^2^) | 24.1±4.0 | 22.7±4.0 | 21.1±4.8 | 23.3±4.3 | 0.143 | |  |
| TG, (mmol/L) | 2.0±1.0 | 1.6±0.7 | 1.1±0.6 | 1.2±0.5 | 0.008 | |  |
| LDL-C, (mmol/L) | 2.5±0.6 | 3.1±0.9 | 2.8±1.2 | 2.9±0.7 | 0.083 | |  |
| FPG, (mmol/L) | 4.90±1.12 | 5.10±0.75 | 4.98±1.20 | 4.81±1.25 | 0.905 | |  |
| Hypertension, n (%) | 10 (90.9) | 22 (95.7) | 13 (86.7) | 15 (100) | 0.461 | |  |
| Antihypertensive drug, n (%) | 6 (54.5) | 17 (73.9) | 6 (40.0) | 4 (26.7) | 0.027 | |  |
| CVD medication, n (%) | 9 (81.8) | 10 (43.5) | 2 (13.3) | 2 (13.3) | 0.001 | |  |
|  | | | | | | |  |
| BMI, body mass index; CVD, cardiovascular disease; FPG, fasting plasma glucose;  LDL-C, low-density lipoprotein cholesterol; TG, triglycerides. | | | | | | | |


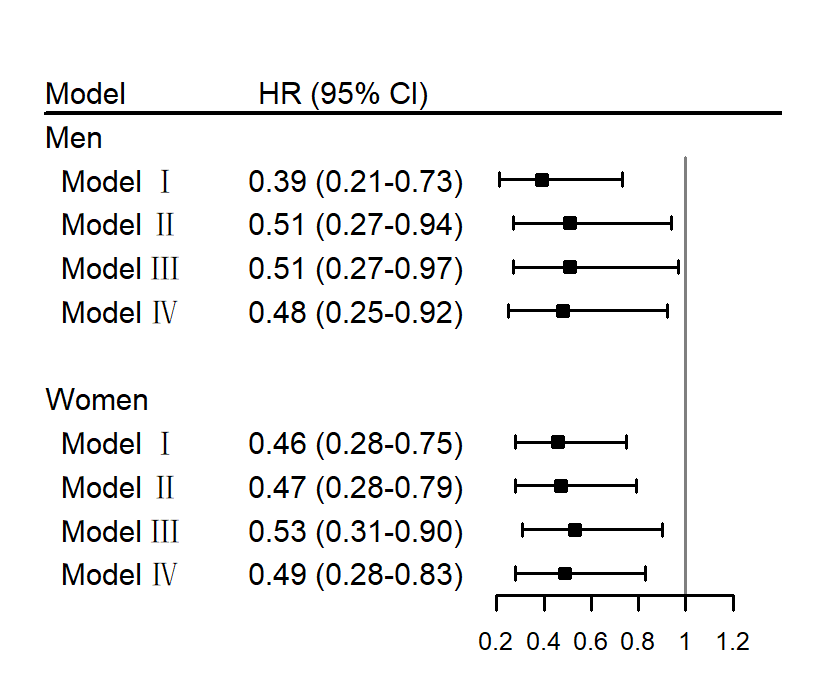


**Supplementary Figure S1.** Hazard Ratio (HR) and 95% confidence intervals (CI) with HDL-C on the risk of T2DM in the subgroup analysis.

Model I: Adjustments were made for gender, age, education, smoking status, alcohol intake, regular exercise, residence and job type.

Model II: Model I along with hypertension, BMI, consumptions of staple foods and eggs.

Model III: Model II along with LDL-C, TG, antihypertensive drug and cardiovascular medication use.

Model IV: Model III along with FPG.
